# FHL2 facilitates LUSC growth and therapy resistance through PI3K/AKT/mTOR activation

**DOI:** 10.1016/j.jbc.2025.110332

**Published:** 2025-06-06

**Authors:** Lingxian Zhang, Dingguo Wang, Lei Zeng, Shiwei Chen, Kunchao Li, Tiankai Yuan, Jing Wang, Xiong Ma, Shuqiang Zhu, Yongbing Wu

**Affiliations:** 1 Department of Cardiothoracic Surgery, The Second Affiliated Hospital, Jiangxi Medical College, Nanchang University, Nanchang, Jiangxi province, China; 2 Department of Pathology, The Second Affiliated Hospital, Jiangxi Medical College, Nanchang University, Nanchang, Jiangxi province, China

**Keywords:** FHL2, LUSC, afatinib, PDK1, c-Jun

## Abstract

Four and a half LIM domain protein 2 (FHL2) plays a key role in tumorigenesis and progression. This study investigated its involvement in lung squamous cell carcinoma (LUSC). Bioinformatics analysis and immunohistochemistry confirmed that FHL2 is significantly upregulated in LUSC tissues and correlates with poor prognosis. Gain and loss experiments demonstrated that FHL2 promotes LUSC cell proliferation, migration, and invasion in vitro, while xenograft models confirmed its role in tumor growth in vivo. Mechanistically, FHL2 interacts with c-Jun and suppresses its ubiquitination, thereby stabilizing the c-Jun protein, upregulating PDK1 expression, and subsequently activating the PAM signaling pathway. Notably, FHL2 overexpression induced afatinib resistance in LUSC cells, and patients with afatinib resistance exhibited high levels of FHL2 expression. Our results demonstrate that FHL2 promotes LUSC progression and induces afatinib resistance by regulating the PAM signaling pathway. FHL2 may serve as a crucial prognostic marker for the survival outcomes of LUSC patients and a promising therapeutic target for their treatment.

Lung cancer ranks as the second most frequently diagnosed cancer and remains the leading cause of cancer-related mortality worldwide, accounting for approximately 2.2 million new cases and 1.8 million deaths in 2020 (1). The 5-year relative survival rate for lung cancer remains low, ranging from 10% to 20% in most countries for diagnoses made between 2010 and 2014, while in the United States, it improved to 25% for cases diagnosed between 2013 and 2019 (1, 2). Non–small cell lung cancer (NSCLC) accounts for approximately 85% of lung cancer cases, with lung squamous cell carcinoma (LUSC) being the second most common subtype, representing 30% of NSCLC cases (3). Thus, LUSC remains a significant global health burden. However, EGFR tyrosine kinase inhibitors (TKIs) and ALK inhibitors are typically not used for LUSC patients due to the absence of activating epidermal growth factor receptor (EGFR) mutations and ALK fusions (4). Although immune checkpoint inhibitors have become a cornerstone of standard treatment strategies for LUSC, their clinical efficacy remains limited, with a relatively low overall response rate (5). Therefore, identifying novel therapeutic targets for LUSC patients is of critical importance.

Four and a half LIM domains protein 2 (FHL2), short for four and a half LIM-only protein 2, comprises four and a half LIM domains and is a member of the FHL protein family. The LIM domain, characterized by a unique double zinc finger motif, is found in proteins that play diverse roles in biological processes, including gene transcription regulation, cytoskeletal organization, cell lineage determination, and organ development (6). FHL2 is ubiquitously expressed in a range of tissues throughout the body, with particularly high expression in cardiac tissue. FHL2 mutation and aberrant expression are linked with cardiac disease, such as dilated cardiomyopathy (7) and hypertrophic cardiomyopathy (8). An increasing number of studies have been conducted to investigate the association between FHL2 and malignant tumors. FHL2 expression is elevated in gastrointestinal cancers, and its suppression has been shown to inhibit the progression of gastric and colon cancer (9). FHL2 interacts with both EGFR and its mutant form EGFRVIII, elevating their expression levels and thereby facilitating glioma development (10). However, FHL2 is frequently downregulated in hepatocellular carcinoma (HCC) and engages with Smad2-4 to regulate gene expression, ultimately inhibiting HCC cell proliferation (11). As a critical adaptor and scaffolding protein, FHL2 exerts its functions through interactions with specific proteins. Its dual roles are determined by whether it associates with oncoproteins or tumor suppressors. What is particularly interesting is that, as of now, the expression levels and function of FHL2 in LUSC remain unexplored.

The PI3K/AKT/mTOR (PAM) pathway is activated by insulin, growth factors, and cytokines, serving as a fundamental signaling cascade in eukaryotic cells that regulates critical processes such as cell survival, growth, and proliferation (12, 13). The PAM pathway is among the most frequently activated signaling pathways in human cancers, with its dysregulation driving tumorigenesis and cancer progression (14). Moreover, hyperactivation of the PAM pathway in cancer often underlies the emergence of treatment resistance (13, 14, 15). Consequently, an increasing number of PI3K-, AKT-, and mTOR-targeted inhibitors are being developed to address therapeutic resistance associated with TKIs (16, 17). 3-Phosphoinositide-dependent protein kinase 1 (PDK1), a key member of the AGC kinase family, plays an essential role in the PAM signaling pathway. PDK1 is activated through its interaction with phosphatidylinositol-3,4,5-trisphosphate (PIP3) *via* the C-terminal pleckstrin homology domain. Upon activation, PDK1 phosphorylates and activates various downstream substrates through its N-terminal serine/threonine kinase domain (the T or activation loop) (18). These substrates include AKT, glycogen synthase kinase 3β (GSK3β), p70 ribosomal protein S6 kinase (p70S6K), and tuberous sclerosis complex 2, which collectively regulate critical cellular processes such as growth, survival, and metabolism (19, 20). Although no PDK1-activating mutations have been identified in tumors to date, PDK1 is frequently overexpressed in various human cancers, where its upregulation drives tumorigenesis and progression (21).

Afatinib, a second-generation oral TKI and pan-ERBB inhibitor, targets all ErbB family members, including EGFR, HER2, HER3, and HER4 (22). The Phase III LUX-Lung 8 trial demonstrated its superiority over erlotinib in improving progression-free and overall survival (OS) in LUSC patients (23). Consequently, the Food and Drug Administration has approved afatinib as a first-line treatment for metastatic LUSC (24). The NCCN Panel's 2022 update (Version 1) recommends afatinib or Osimertinib as preferred first-line therapies for metastatic NSCLC with specific EGFR mutations, such as S768I, L861Q, and G719X (25). Unfortunately, the clinical use of afatinib is constrained by its toxicity profile and the development of resistance (26). Consequently, identifying more effective therapeutic options is a critical unmet need for LUSC patients.

In this study, we found that FHL2 was upregulated in LUSC tissues, with higher FHL2 levels correlating with poor prognosis. Functional assays and xenograft models demonstrated FHL2's role in promoting LUSC cell proliferation, migration, invasion, and metastasis *in vitro* and *in vivo*. Mechanistically, FHL2 was identified as a coactivator of c-Jun through immunoprecipitation (IP) and proximity ligation assay (PLA). Moreover, FHL2 overexpression induced afatinib resistance, suggesting its potential as a therapeutic target for LUSC patients.

## Results

### Bioinformatics analysis reveals upregulation of FHL2 expression in NSCLC

**Figure 1:**
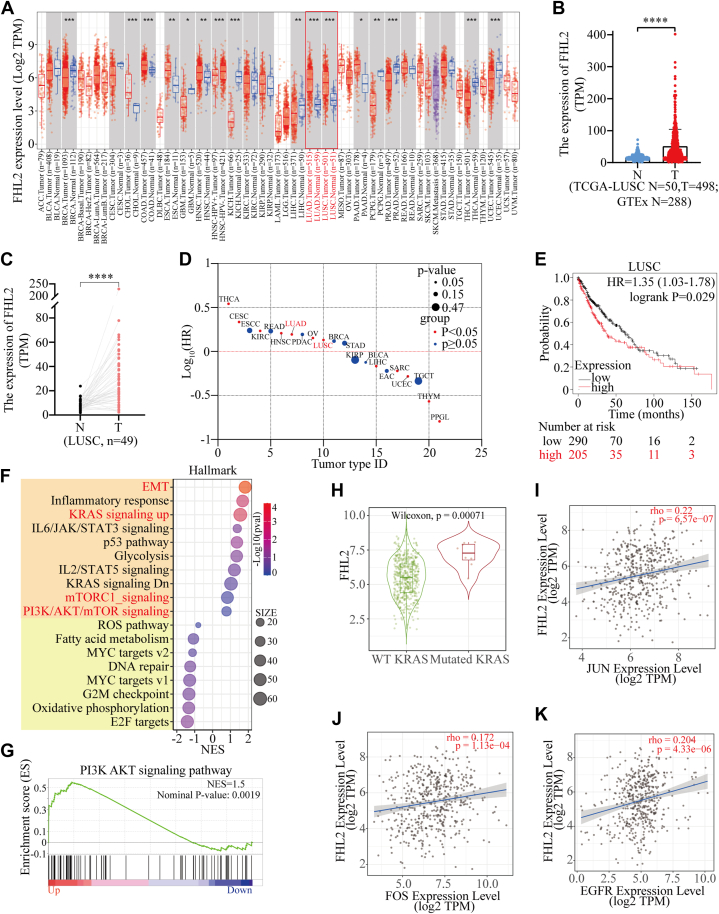
**Pan-cancer analysis of FHL2 expression and its implications.***A*, FHL2 expression in various tumor and normal tissues from the TIMER 2.0 database. *B*, FHL2 expression in LUSC tissues and normal lung tissues based on data from the TCGA and GTEx databases. Data are presented as mean ± SD. *C*, FHL2 expression in 49 paired LUSC and normal lung tissues from TCGA. The data were assessed with paired *t* test. *D*, bubble plot of survival curves across various malignant cancers based on data from Kaplan–Meier plotter. *E*, analysis of FHL2 expression and overall survival (OS) in LUSC based on TCGA data. *F*, bubble plot showing signaling pathways from GSEA analysis of TCGA data, with patients grouped into FHL2 high and low expression groups. The cutoff value is the same as in *E*. *G*, enrichment plot of the PI3K/AKT signaling pathway from GSEA analysis. *H*, differential FHL2 expression between KRAS WT and mutant groups in LUSC from the TIMER 2.0 database. *I*–*K*, correlation analysis of FHL2 and JUN, FOS, EGFR expression from the TIMER 2.0 database. ∗*p* < 0.05, ∗∗*p* < 0.01, ∗∗∗*p* < 0.001, ∗∗∗∗*p* < 0.0001. LUSC, lung squamous cell carcinoma; FHL2, four and a half LIM domains protein 2; EGFR, epidermal growth factor receptor; TCGA, The Cancer Genome Atlas; GSEA, Gene Set Enrichment Analysis; Genotype-Tissue Expression.

**Figure S1:**
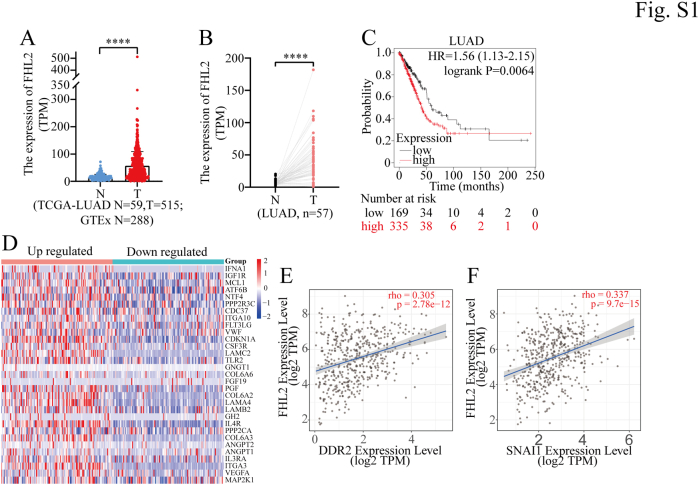


The expression of FHL2 is significantly elevated in certain malignant tumors, including cholangiocarcinoma (CHOL), colon adenocarcinoma (COAD), esophageal carcinoma (ESCA), head and neck squamous cell carcinoma (HNSC), lung adenocarcinoma (LUAD), LUSC, and pancreatic adenocarcinoma (PAAD). Conversely, it is markedly reduced in other types of malignancies as analyzed using the TIMER 2.0 database (http://timer.cistrome.org/), such as glioblastoma multiforme (GBM), kidney chromophobe (KICH), liver hepatocellular carcinoma (LIHC), pheochromocytoma and paraganglioma (PCPG), prostate adenocarcinoma (PRAD), thyroid carcinoma (THCA), and uterine corpus endometrial carcinoma (UCEC) (Fig. 1*A*). (Abbreviations of tumor types are provided at the end of this article). In addition, we integrated data from The Cancer Genome Atlas (TCGA) and Genotype-Tissue Expression (GTEx) databases, revealing that FHL2 expression was significantly elevated in LUSC and LUAD compared to normal lung tissues (Fig. 1*B* & S1*A*). We then analyzed FHL2 expression in 49 paired LUSC and 57 paired LUAD tissue samples, along with adjacent lung tissues, from the TCGA database. The results demonstrated a significant upregulation of FHL2 in both LUSC (Fig. 1*C*) and LUAD (Fig. S1*B*). For prognosis, high FHL2 expression was associated with poor OS in patients with THCA, cervical squamous cell carcinoma and endocervical adenocarcinoma (CESC), kidney renal clear cell carcinoma (KIRC), HNSC, LUAD, ovarian serous cystadenocarcinoma (OV), and LUSC. In contrast, patients with elevated FHL2 expression exhibited favorable OS in LIHC, sarcoma (SARC), UCEC, thymoma (THYM), and PCPG (Fig. 1*D*). The OS curves revealed a significant difference between the high and low FHL2 expression groups in both LUSC (Fig. 1*E*) and LUAD (Fig. S1*C*). Gene Set Enrichment Analysis (GSEA) was performed to investigate the function of FHL2 in LUSC using TCGA data. The bubble plots illustrate the significantly altered signaling pathways in both the high and low FHL2 expression groups (Fig. 1*F*). Notably, the PAM and mTOR signaling pathways were upregulated in the FHL2 high expression group. The enrichment plot further demonstrated that the PI3K/AKT signaling pathway was upregulated in the FHL2 high expression group (Fig. 1*G*). The heatmap illustrates the differential expression of core genes in the PI3K/AKT signaling pathway between the FHL2 high and low groups in LUSC (Fig. S1*D*). Finally, coexpression analyses were performed using the TIMER 2.0 database to examine the relationship between FHL2 and several oncogenes. FHL2 expression was found to be upregulated in KRAS mutant LUSC patients (Fig. 1*H*). Moreover, FHL2 expression showed a positive correlation with the expression of JUN (Fig. 1*I*), FOS (Fig. 1*J*), EGFR (Fig. 1*K*), DDR2 (Fig. S1*E*), and SNAIL1 (Fig. S1*F*). These data indicate that FHL2 expression is upregulated in LUSC, and patients with high FHL2 expression tend to have a poorer prognosis. Mechanistically, FHL2 may promote LUSC progression by upregulating the PAM signaling pathway.

### FHL2 protein is abnormally upregulated in LUSC and correlates with poor prognosis

**Figure 2:**
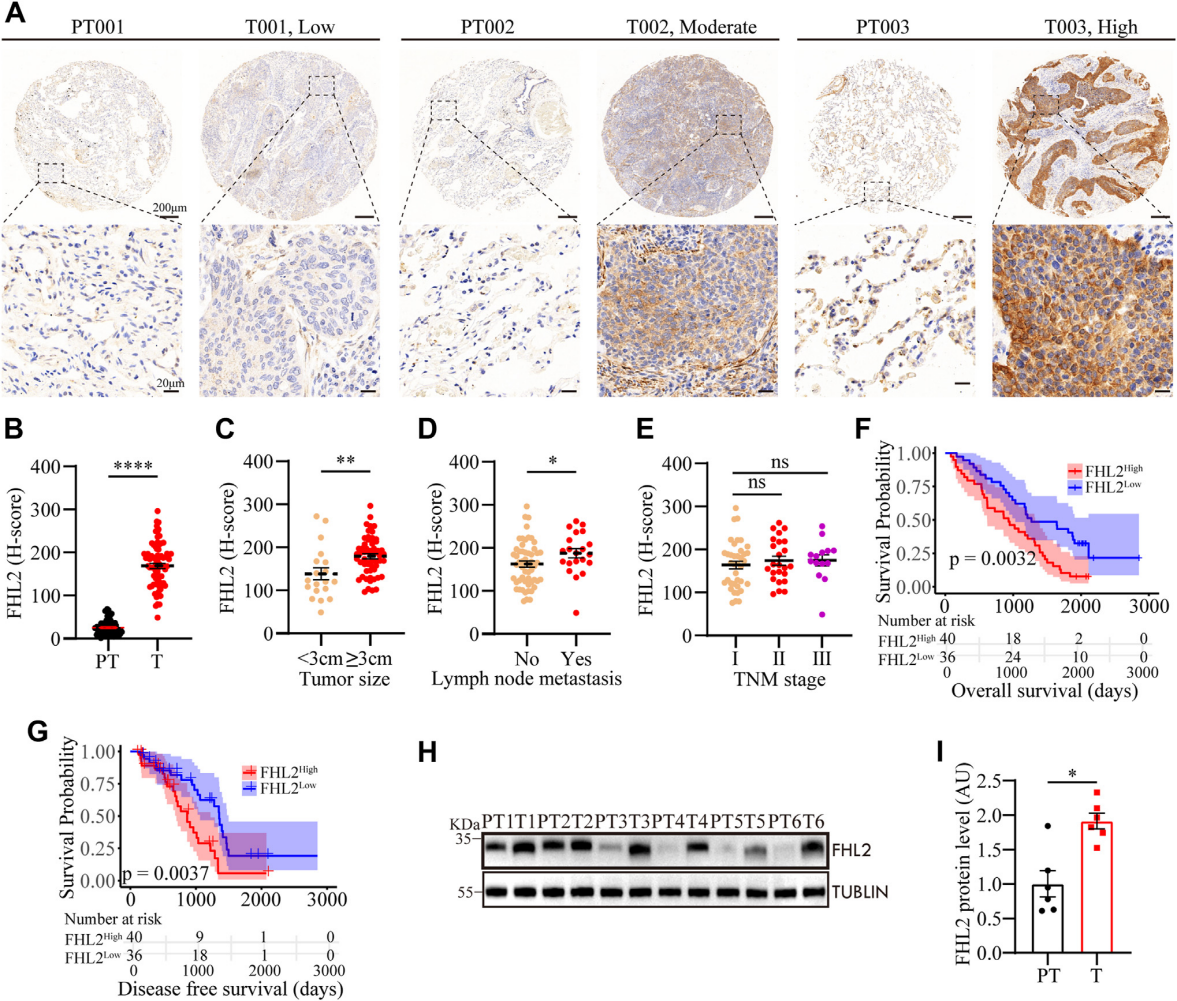
**FHL2 protein levels in 76 LUSC samples and their association with clinicopathologic characteristics.***A*, representative images of FHL2 IHC staining in a TMA comprising 76 LUSC tissues and adjacent normal lung tissues. *B*, scatter plot showing the quantification of IHC staining, with H-scores representing FHL2 protein levels. *C*, scatter plot illustrating the differential analysis of FHL2 levels based on tumor size. *D*, scatter plot depicting the variation in FHL2 levels relative to lymph node metastasis states. *E*, scatter plot presenting the association between FHL2 levels and TNM stage. *F*, prognostic analysis of overall survival in relation to FHL2 levels using Kaplan–Meier plotter. *G*, Kaplan–Meier analysis of disease-free survival (DFS) based on FHL2 levels. *H* and *I*, WB analysis of FHL2 protein levels in six paired LUSC tissues and adjacent normal lung tissues, with quantification results shown. The data were assessed with a two-tailed unpaired *t* test. ∗*p* < 0.05, ∗∗*p* < 0.01, ∗∗∗∗*p* < 0.0001, ns: no significant. LUSC, lung squamous cell carcinoma; FHL2, four and a half LIM domains protein 2; IHC, immunohistochemistry; TMA, tissue microarray; TNM, tumour, node, metastasis; WB, Western blotting.

To further investigate FHL2 expression and its association with clinicopathological characteristics, immunohistochemistry (IHC) staining was performed on tissue microarrays (TMAs) comprising 76 LUSC tissues and adjacent normal lung tissues (Fig. 2*A*). Quantification was carried out using the Automatic H-score Quantification (AHSQ) online tool, revealing that FHL2 protein level was significantly elevated in LUSC tissues compared to adjacent normal lung tissues (Fig. 2*B*). The association between FHL2 levels and various clinicopathologic parameters was evaluated and summarized in Table 1. Patients with larger tumors (>3 cm) or lymph node metastasis exhibited significantly higher FHL2 levels compared to those with smaller tumors or no metastasis (Fig. 2, *C* and *D*). Patients with advanced TNM stages tended to have higher FHL2 levels, although the difference was not statistically significant (Fig. 2*E*). We further investigated the association between FHL2 levels and LUSC prognosis. The analysis revealed that patients with elevated FHL2 levels had significantly shorter OS (*p* = 0.0032, Fig. 2*F*) and disease-free survival (DFS) (*p* = 0.0037, Fig. 2*G*) compared to those with lower FHL2 levels. Univariate analysis demonstrated that tumor stage, lymph node metastasis (*p* = 0.041), and FHL2 level (*p* = 0.012) were significantly associated with OS (Table 2). Similarly, tumor stage, lymph node metastasis (*p* = 0.009), and FHL2 level (*p* = 0.015) showed significant associations with DFS (Table 3). Multivariate analysis further identified tumour, node, metastasis stage and FHL2 level (HR: 1.470, 95% CI: 1.093–1.978, *p* = 0.011) as independent prognostic factors for OS (Table 2), while tumor stage (III *versus* I) and FHL2 level (HR: 1.545, 95% CI: 1.078–2.213, *p* = 0.018) were independent prognostic factors for DFS (Table 3). To further validate FHL2 levels, we selected six paired LUSC and adjacent normal lung tissues for Western blotting (WB) analysis. The results were consistent with the IHC staining, showing a significant upregulation of FHL2 levels in LUSC tissues (Fig. 2, *H* and *I*). These data validate the upregulation of FHL2 in LUSC tissues and highlight its significant association with clinicopathologic features and prognosis. Elevated FHL2 levels correlate with poor OS and DFS, suggesting that FHL2 may serve as a potential biomarker for the progression and prognosis of LUSC.

**Table 1:** Correlations between FHL2 level and clinical characteristics in 76 LUSC patients

Clinicopathologic parameters	No. of cases	FHL2 expression level		*p* Value
		Low	High	
Age				
≥60 years	56	24	32	
<60 years	20	12	8	0.290
Gender				
Male	75	36	39	
Female	1	0	1	1.000
Smoking history				
Smokers	15	7	8	
Nonsmokers	61	29	32	1.000
Tumor stage				
I	38	18	20	
II	24	14	10	
III	14	4	10	0.208
Lymph node metastasis				
Yes	21	6	15	
No	55	30	25	0.077
Tumor size				
<3 cm	19	14	5	
≥3 cm	57	22	35	0.017
Differentiation				
Well and moderate	57	30	27	
Poor	19	6	13	0.185

LUSC, lung squamous cell carcinoma; FHL2, four and a half LIM domains protein 2.

**Table 2:** Univariate and multivariate analyses of factors associated with overall survival

Factors	OS					
	Univariate			Multivariate		
	HR	95% CI	*p* Value	HR	95% CI	*p* Value
Age (≥60 *versus* < 60) years	0.752	0.430–1.317	0.320			NA
Gender (Male *versus* Female)	0.223	0.029–1.690	0.147			NA
Smoking history (smokers *versus* nonsmokers)	0.773	0.423–1.413	0.403			NA
Tumor stage (II *versus* I)	2.151	1.222–3.789	0.008	2.476	1.287–4.763	0.007
Tumor stage (III *versus* I)	2.746	1.407–5.360	0.003	3.802	1.658–8.718	0.002
Lymph node metastasis (Yes *versus* No)	1.763	1.025–3.035	0.041	0.643	0.312–1.326	0.232
Tumor size (≥3 cm *versus* < 3 cm)	1.863	0.986–3.519	0.055			NA
Differentiation (poor *versus* well and moderate)	1.108	0.618–1.986	0.730			NA
FHL2 level (high *versus* low)	1.411	1.078–1.847	0.012	1.470	1.093–1.978	0.011

NA, not adopted; CI, confidence interval; HR, hazard ratio; FHL2, four and a half LIM domains protein 2.

**Table 3:** Univariate and multivariate analyses of factors associated with disease-free survival

Factors	OS					
	Univariate			Multivariate		
	HR	95% CI	*p* Value	HR	95% CI	*p* Value
Age (≥60 *versus* < 60) years	0.612	0.313–1.199	0.152			NA
Gender (male *versus* female)	/	/	/			/
Smoking history (smokers *versus* nonsmokers)	1.101	0.510–2.380	0.806			NA
Tumor stage (II *versus* I)	2.336	1.157–4.718	0.018	2.315	0.968–5.536	0.059
Tumor stage (III *versus* I)	3.271	1.514–7.068	0.003	3.311	1.285–8.531	0.013
Lymph node metastasis (Yes *versus* No)	2.357	1.237–4.494	0.009	0.990	0.419–2.337	0.982
Tumor size (≥3 cm *versus* < 3 cm)	1.613	0.792–3.284	0.188			NA
Differentiation (poor *versus* well and moderate)	1.596	0.831–3.065	0.161			NA
FHL2 level (high *versus* low)	1.509	1.083–2.101	0.015	1.545	1.078–2.213	0.018

NA, not adopted; FHL2, four and a half LIM domains protein 2; CI, confidence interval; OS, overall survival; HR, hazard ratio.

### FHL2 promotes the progression of LUSC *in vitro* and *in vivo*

**Figure 3:**
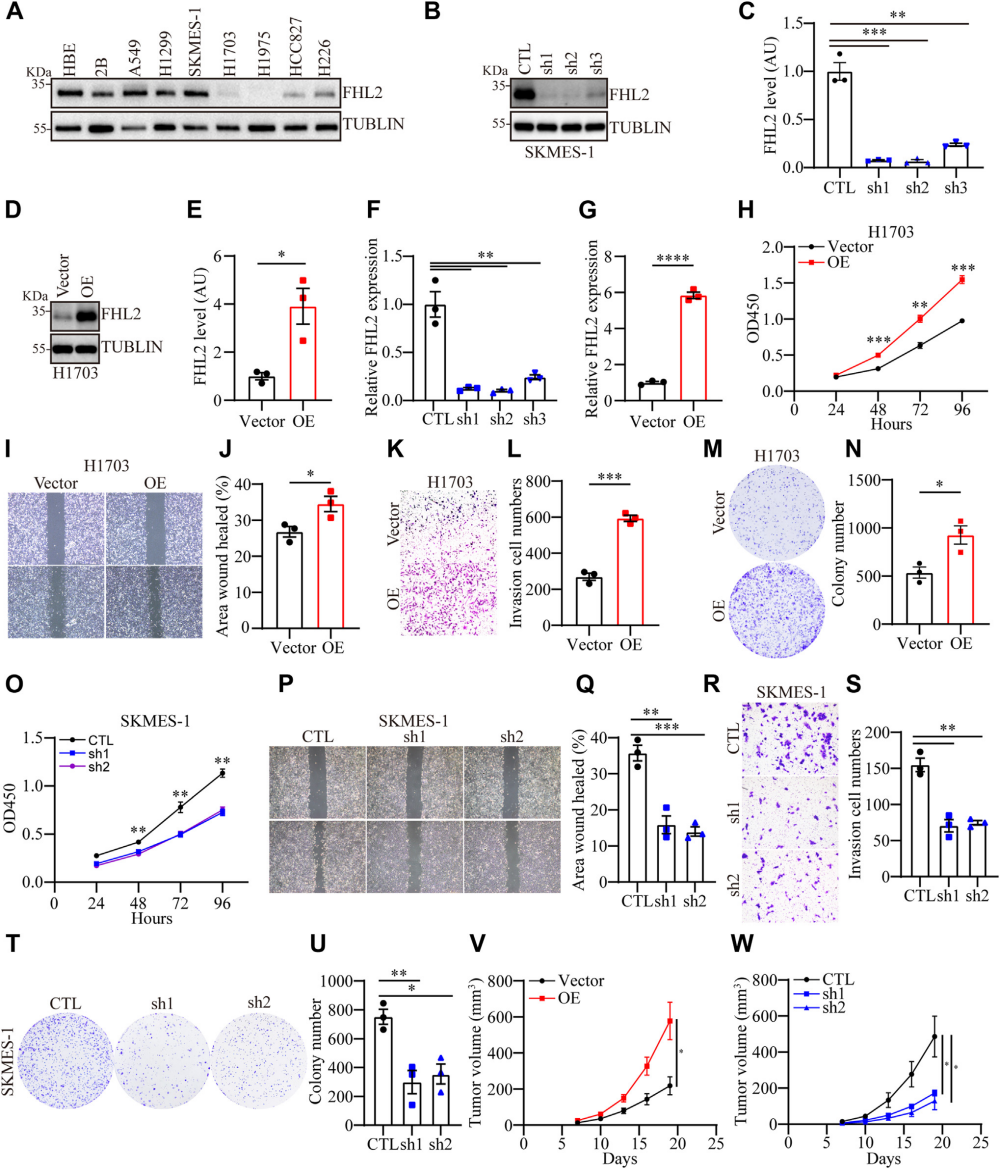
**Upregulation of FHL2 promotes LUSC progression *in vitro*** and *in vivo*. *A*, WB analysis of FHL2 levels in human bronchial epithelial cells (HBE and Beas-2B) and seven NSCLC cell lines (A549, NCI-H1299, SK-MES-1, NCI-H1703, NCI-H1975, HCC827, and NCI-H226). *B* and *C*, WB analysis of the knockdown efficiency of FHL2 in SK-MES-1 cells and the quantification. *D* and *E*, WB analysis of the overexpression efficiency of FHL2 in H1703 cells and the quantification. *F* and *G*, qPCR assessment of the knockdown efficiency of FHL2 in SK-MES-1 cells and the overexpression efficiency of FHL2 in H1703 cells. *H*, the proliferation ability was assessed by CCK-8 assay in H1703 cells with FHL2 overexpression. *I* and *J*, wound healing assay was performed to evaluate the migration of H1703 cells with FHL2 overexpression, with quantification shown in (*J*). *K* and *L*, Matrigel Transwell assay was conducted to evaluate the invasion of H1703 cells with FHL2 overexpression, with quantification provided in (*L*). *M* and *N*, the effect of FHL2 overexpression on clonogenic potential was measured by plate colony formed assay in H1703 cells, with quantification provided in (*N*). *O*, the proliferation ability was assessed by CCK-8 assay in SK-MES-1 cells with FHL2 knockdown. *P* and *Q*, wound healing assay was performed in SK-MES-1 cells with FHL2 knockdown, with quantification provided. *R* and *S*, the invasion of SK-MES-1 cells with FHL2 knockdown was assessed using Matrigel Transwell assay, with quantification results shown. *T* and *U*, FHL2 overexpression influenced the number of colonies formed by SK-MES-1 cells, with quantification results provided. *V* and *W*, xenograft models using SK-MES-1 and H1703 cells with FHL2 knockdown or overexpression were established to evaluate the role of FHL2 in promoting LUSC progression *in vivo*. The data were assessed with a two-tailed unpaired *t* test. ∗*p* < 0.05, ∗∗*p* < 0.01, ∗∗∗*p* < 0.001, ∗∗∗∗*p* < 0.0001. LUSC, lung squamous cell carcinoma; FHL2, four and a half LIM domains protein 2; NSCLC, non–small cell lung cancer; qPCR, quantitative PCR; WB, Western blotting; CCK-8, Cell Counting Kit-8.

**Figure S2:**
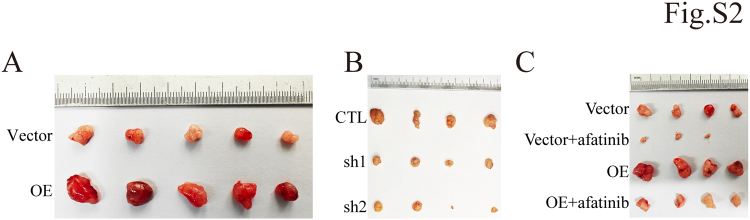


To further explore the role of FHL2, we planned to perform gain- and loss-of-function experiments both *in vivo* and *in vitro*. Initially, we assessed FHL2 levels in human bronchial epithelial cells (HBE and Beas-2B) and seven NSCLC cell lines (A549, NCI-H1299, SK-MES-1, NCI-H1703, NCI-H1975, HCC827, and NCI-H226) using WB analysis. The results revealed that FHL2 was highly expressed in A549, H1299, and SK-MES-1 cells, while it exhibited low expression in NCI-H1703 and NCI-H1975 cells. In addition, intermediate expression levels were observed in HCC827 and NCI-H226 cells (Fig. 3*A*). Hence, we selected two LUSC cell lines, NCI-H1703 and SK-MES-1, for FHL2 overexpression and knockdown experiments. The efficiency of FHL2 overexpression and knockdown was validated using WB (Fig. 3, *B*–*E*) and quantitative PCR (qPCR) (Fig. 3, *F* and *G*). Three short hairpin RNAs (shRNAs) were evaluated, and the two achieving the most effective knockdown (shRNA1 and shRNA2) were chosen for further experiments. FHL2 overexpression markedly augmented the proliferative capacity (Fig. 3*H*), migratory ability (Fig. 3, *I* and *J*), invasive potential (Fig. 3, *K* and *L*), and clonogenic efficiency (Fig. 3, *M* and *N*) in H1703 cells. In contrast, FHL2 knockdown significantly attenuated these attributes, including proliferation (Fig. 3*O*), migration (Fig. 3, *P* and *Q*), invasion (Fig. 3, *R* and *S*), and clonogenic capacity (Fig. 3, *T* and *U*) in SK-MES-1 cells. To further validate the functional role of FHL2 *in vivo*, xenograft models were established using H1703 and SK-MES-1 cells with stable FHL2 overexpression or knockdown in male BALB/c-nu/nu mice. Tumors derived from FHL2-overexpressing cells exhibited significantly larger volumes and accelerated growth rates compared to those from control cells. Conversely, FHL2 knockdown led to a marked reduction in tumor size and a significant inhibition of tumor growth (Fig. 3, *V* and *W* and S2, *A* and *B*). These findings collectively demonstrate that FHL2 plays a pivotal role in LUSC tumor progression by promoting proliferation, migration, invasion, and clonogenic capacity.

### Differential expression of FHL2 may regulate the PAM signaling pathway

**Figure 4:**
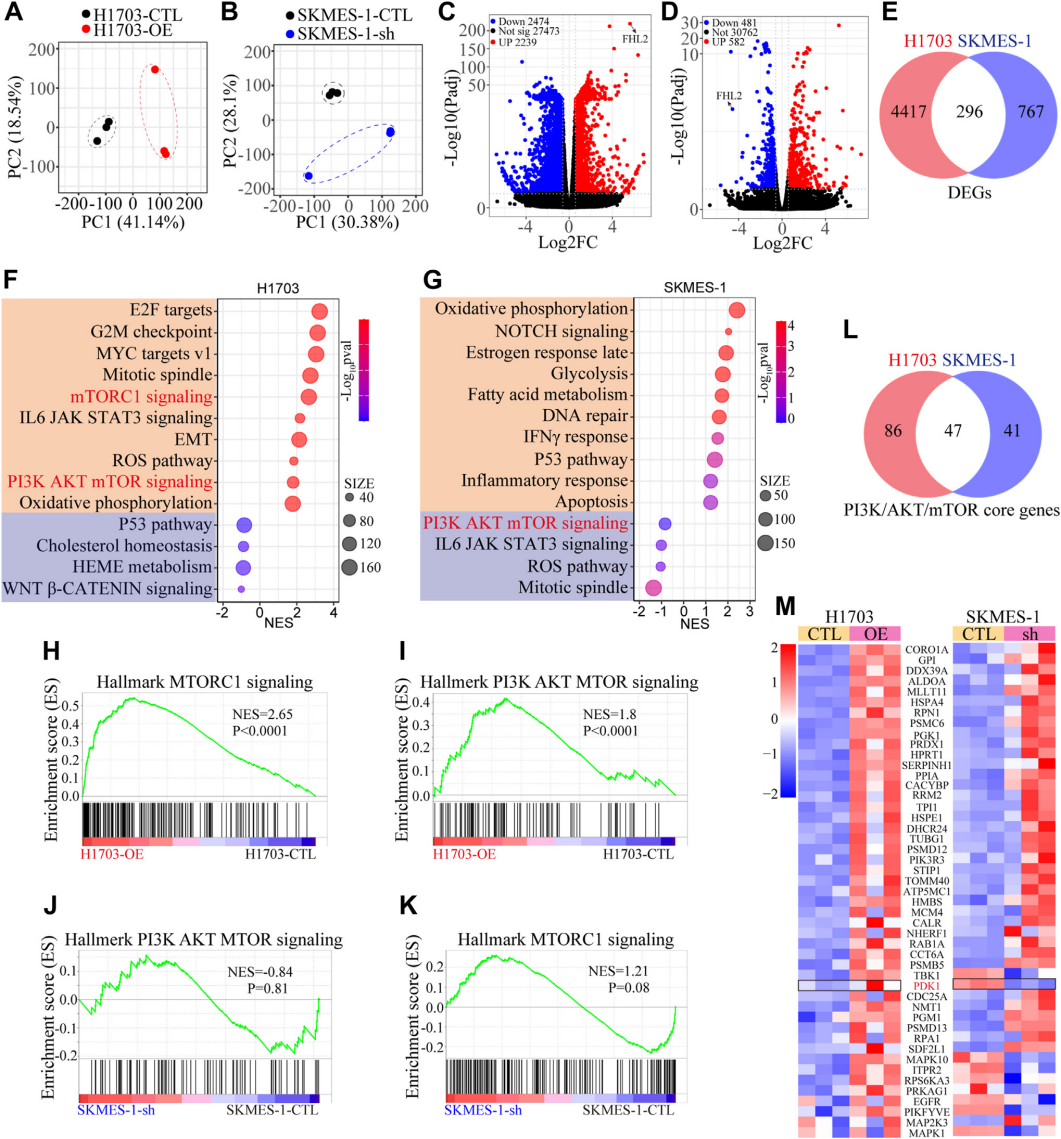
**Bulk RNA-seq analysis of H1703 and SK-MES-1 cells with FHL2 overexpression or knockdown**. *A* and *B*, PCA plots of bulk RNA-seq data from H1703 cells with FHL2 overexpression and SK-MES-1 cells with FHL2 knockdown. *C* and *D*, volcano plots displaying differentially expressed genes (DEGs) resulting from FHL2 overexpression or knockdown. *E*, venn diagram illustrating the overlap of DEGs in H1703 and SK-MES-1 cells following FHL2 overexpression or knockdown. *F* and *G*, bubble plots presenting GSEA results analyzed using hallmark gene sets. *H* and *I*, enrichment plots of the mTOR and PI3K/AKT/mTOR signaling pathways in SK-MES-1 cells with FHL2 knockdown. *J* and *K*, enrichment plots of the mTOR and PI3K/AKT/mTOR signaling pathways in H1703 cells with FHL2 overexpression. *L*, venn diagram showing the intersection of core genes from the PI3K/AKT/mTOR signaling pathway in H1703 and SK-MES-1 cells with FHL2 overexpression or knockdown. *M*, heatmap showing the expression of 47 core genes from PI3K/AKT/mTOR signaling pathway. FHL2, four and a half LIM domains protein 2; GSEA, Gene Set Enrichment Analysis; PCA, principal component analysis.

**Figure S3:**
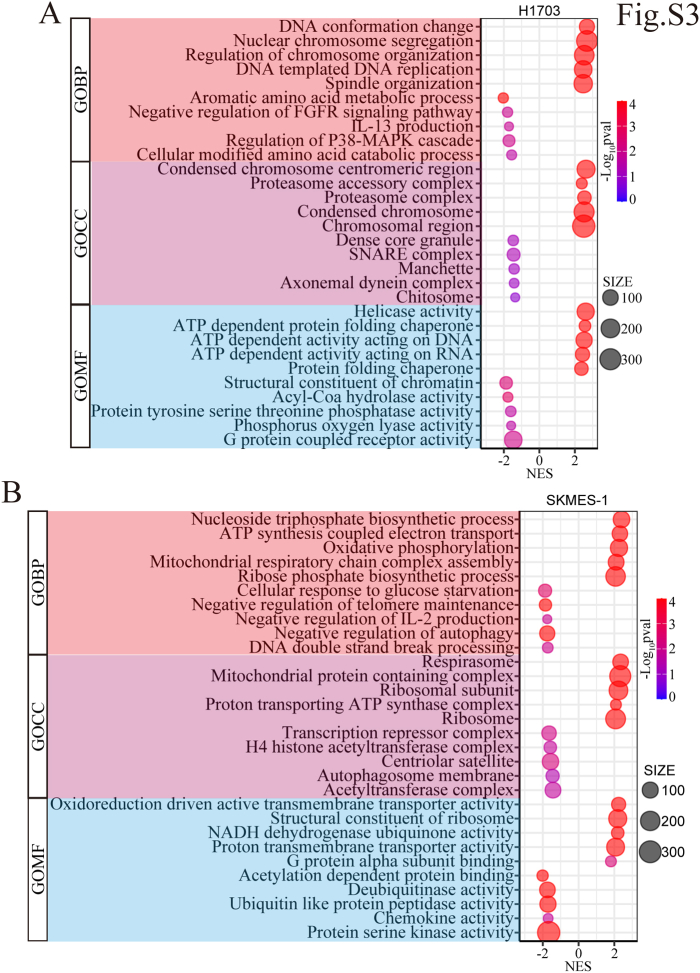


To uncover the underlying mechanisms, bulk RNA sequencing (RNA-seq) was conducted on RNA extracted from H1703 and SK-MES-1 cells with FHL2 overexpression or knockdown. The principal component analysis revealed that the first two principal components (PC1 and PC2) accounted for 59.68% of the total variance in H1703 cells (PC1: 41.14% and PC2: 18.54%) (Fig. 4*A*), and 58.48% of the total variance in SK-MES-1 cells (PC1: 30.38% and PC2: 28.1%) (Fig. 4*B*). The distinct separation between groups suggests differential gene expression profiles, which may be driven by FHL2-mediated effects. The volcano plots demonstrate the significance and expression changes of differentially expressed genes (|Fold Change| > 1.5, *p* < 0.05), with upregulated genes depicted in red, downregulated genes in blue, and non-significant genes in black (Fig. 4, *C* and *D*). A Venn diagram illustrates the overlap of differentially expressed genes (296 genes) between H1703 and SK-MES-1 cells (Fig. 4*E*). GSEA was conducted using the gene matrix from H1703 and SK-MES-1 cells. The bubble plots depicting the most significantly altered signaling pathways revealed that the mechanistic target of rapamycin complex 1 (mTORC1) and PAM pathways were notably upregulated in H1703 cells with FHL2 overexpression, whereas the PAM pathway was downregulated in SK-MES-1 cells with FHL2 knockdown (Fig. 4, *F* and *G*). In addition, the enrichment plots (Fig. 4, *H*–*K*) clearly illustrate the changes in the mTORC1 and PAM signaling pathways. Next, the core genes of the PAM pathway from H1703 and SK-MES-1 cells were used to generate a Venn diagram (Fig. 4*L*). The differential expression of the 47 overlapping genes is presented in the heatmap (Fig. 4*M*), which highlights that PDK1 is upregulated in H1703 cells with FHL2 overexpression and downregulated in SK-MES-1 cells with FHL2 knockdown. Gene Ontology analyses were also performed, and the results are presented in the bubble plots (Fig. S3*, A and B*). Bulk RNA-seq analysis revealed distinct FHL2-mediated gene expression profiles in H1703 and SK-MES-1 cells, highlighting differential regulation of the PAM pathway and its core genes, including PDK1, as well as significant changes in mTORC1 signaling and Gene Ontology terms associated with FHL2 activity. These findings suggest that FHL2 may promote LUSC progression through the PAM pathway.

### FHL2 acts as a coactivator of c-Jun to regulate PDK1 expression

**Figure 5:**
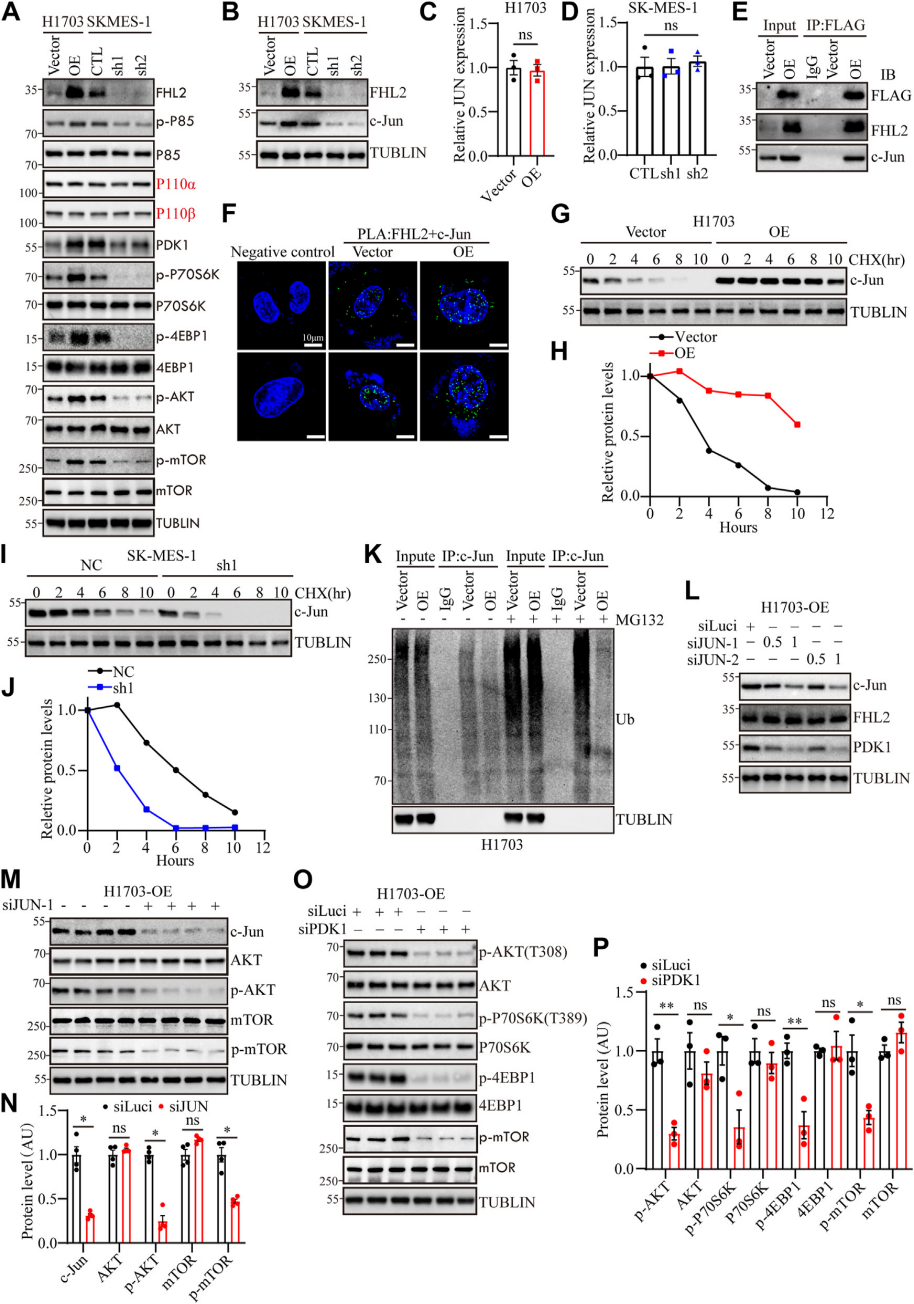
**FHL2 functions as a coactivator of c-Jun to regulate PDK1 expression and the PI3K/AKT/mTOR signaling pathway**. *A*, WB was used to detect PDK1 levels and the PI3K/AKT/mTOR signaling pathway in H1703 and SK-MES-1 cells with altered FHL2 expression. *B*, WB was performed to assess c-Jun expression in H1703 and SK-MES-1 cells with altered FHL2 levels. *C* and *D*, qPCR was conducted to detect JUN mRNA expression in H1703 and SK-MES-1 cells with altered FHL2 levels. *E*, the interaction between FHL2 and c-Jun was detected by IP in H1703 cells. *F*, PLA was performed to examine the interaction between FHL2 and c-Jun in H1703 cells. *G*, WB of H1703 vector control and FHL2-OE cells treated with CHX (50 μM) for indicated times to assess c-Jun stability. *H*, quantification of c-Jun protein levels in (*G*), normalized to TUBLIN. *I*, WB of SK-MES-1 control and FHL2-sh1 cells treated with CHX (50 μM) for indicated times to assess c-Jun stability. *J*, quantification of c-Jun protein levels in (*I*), normalized to TUBLIN. *K*, H1703 vector control and FHL2-OE cells were treated with a proteasome inhibitor MG132 (5 μM) for 6 h, followed by IP and subsequent WB analysis. *L*, PDK1 expression in H1703-OE cells with JUN knockdown was assessed by WB. *M*, WB analysis was performed to evaluate the PI3K/AKT/mTOR signaling pathway activity in H1703-OE cells following JUN knockdown. *N*, the quantification of M. *O* and *P*, PI3K/AKT/mTOR signaling pathway in H1703-OE cells with PDK1 knockdown was assessed by WB, with quantification provided. The data were assessed with multiple *t* test. ∗*p* < 0.05, ∗∗*p* < 0.01, ns: no significant. FHL2, four and a half LIM domains protein 2; qPCR, quantitative PCR; PDK1, 3-Phosphoinositide-dependent protein kinase 1; IP, immunoprecipitation; PLA, proximity ligation assay; CHX, cycloheximide; IP, immunoprecipitation; WB, Western blotting.

**Figure S4:**
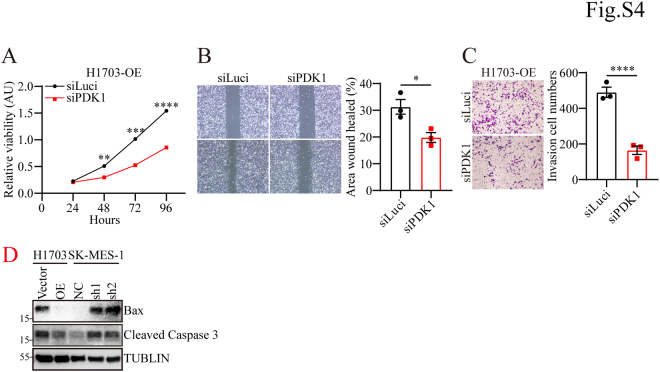


To validate the hypothesis that FHL2 promotes LUSC progression by regulating the PAM pathway, WB analysis was performed. The results demonstrated that FHL2 overexpression increased PDK1 levels and activated p85, AKT, mTOR, P70S6K, and 4EBP1, whereas FHL2 knockdown reduced PDK1 levels and suppressed the PAM pathway (Fig. 5*A*). c-Jun, a component of the activator protein-1 (AP-1) complex, can be induced by extracellular signals and functions as a transcription factor to mediate, amplify, and integrate diverse signaling pathways involved in tissue development and disease (27). JUN, an oncogene highly expressed in various tumors, plays a key role in mediating proliferation, migration, invasion, and epithelial-mesenchymal transition (28). Previous study has shown that FHL2 functions as an inducible coactivator of activator protein-1, significantly enhancing Fos- and Jun-dependent transcription, and identified c-Jun's Ser-63/Ser-73 residues as critical for FHL2 binding (29). Moreover, studies have shown that c-Jun functions as a transcriptional regulator of PDK1 in melanoma (30) and normal bronchial epithelial cells (31), promoting cell proliferation. Hence, we hypothesize that FHL2 may act as a coactivator of c-Jun, regulating PDK1 transcription in LUSC. The WB results confirmed that FHL2 overexpression upregulated c-Jun expression, while FHL2 knockdown decreased c-Jun expression (Fig. 5*B*). However, JUN mRNA levels remained unchanged regardless of FHL2 overexpression in H1703 cells (Fig. 5*C*) or FHL2 knockdown in SK-MES-1 cells (Fig. 5*D*). These results suggest that FHL2 may regulate c-Jun expression through posttranscriptional modifications. IP analysis validated the interaction between FHL2 and c-Jun in H1703 cells (Fig. 5*E*). Furthermore, PLA revealed that FHL2 and c-JUN primarily interact in the nucleus, though their interaction is also detectable in the cytoplasm (Fig. 5*F*). As shown in the cycloheximide (CHX) chase assay, c-Jun protein levels progressively declined over time in H1703 vector control cells, whereas FHL2 overexpression markedly attenuated c-Jun degradation, suggesting that FHL2 stabilizes c-Jun protein (Fig. 5, *G* and *H*). Conversely, in SK-MES-1 cells, knockdown of FHL2 accelerated the degradation of c-Jun, further supporting the role of FHL2 in maintaining c-Jun protein stability (Fig. 5, *I* and *J*). Moreover, the results of IP showed that FHL2 overexpression inhibits the ubiquitination of c-Jun (Fig. 5*K*). Subsequently, we employed varying concentrations of small interfering RNAs (siRNAs) to knock down JUN expression in H1703 cells overexpressing FHL2. WB analysis demonstrated that JUN knockdown led to a reduction in PDK1 expression (Fig. 5*L*). Following JUN knockdown, both p-AKT and p-mTOR levels were markedly reduced (Fig. 5, *M* and *N*), indicating decreased AKT and mTOR activity. Furthermore, PDK1 was depleted using siPDK1 in H1703 cells with FHL2 overexpression, and the activation of the PAM pathway was assessed. WB results indicated that PDK1 knockdown attenuated the PAM pathway (Fig. 5, *O* and *P*). The rescue experiments demonstrated that PDK1 knockdown significantly impaired the proliferation (Fig. S4*A*), migration (Fig. S4*B*), and invasion (Fig. S1*C*) abilities of H1703 cells overexpressing FHL2. These rescue experiments conclusively established that FHL2 functions as a coactivator of c-Jun to regulate PDK1 expression, thereby modulating the PAM signaling pathway. In summary, these results demonstrate that FHL2 promotes LUSC progression by acting as a coactivator of c-Jun, regulating PDK1 transcription, and modulating the PAM pathway, with FHL2 overexpression activating key signaling molecules, while FHL2 knockdown inhibits this pathway. What is interesting is that FHL2 overexpression inhibits apoptosis in H1703 cells and FHL2 knockdown promotes apoptosis in SK-MES-1 cells (Fig. S1*D*).

### FHL2 overexpression induces resistance to afatinib therapy

**Figure 6:**
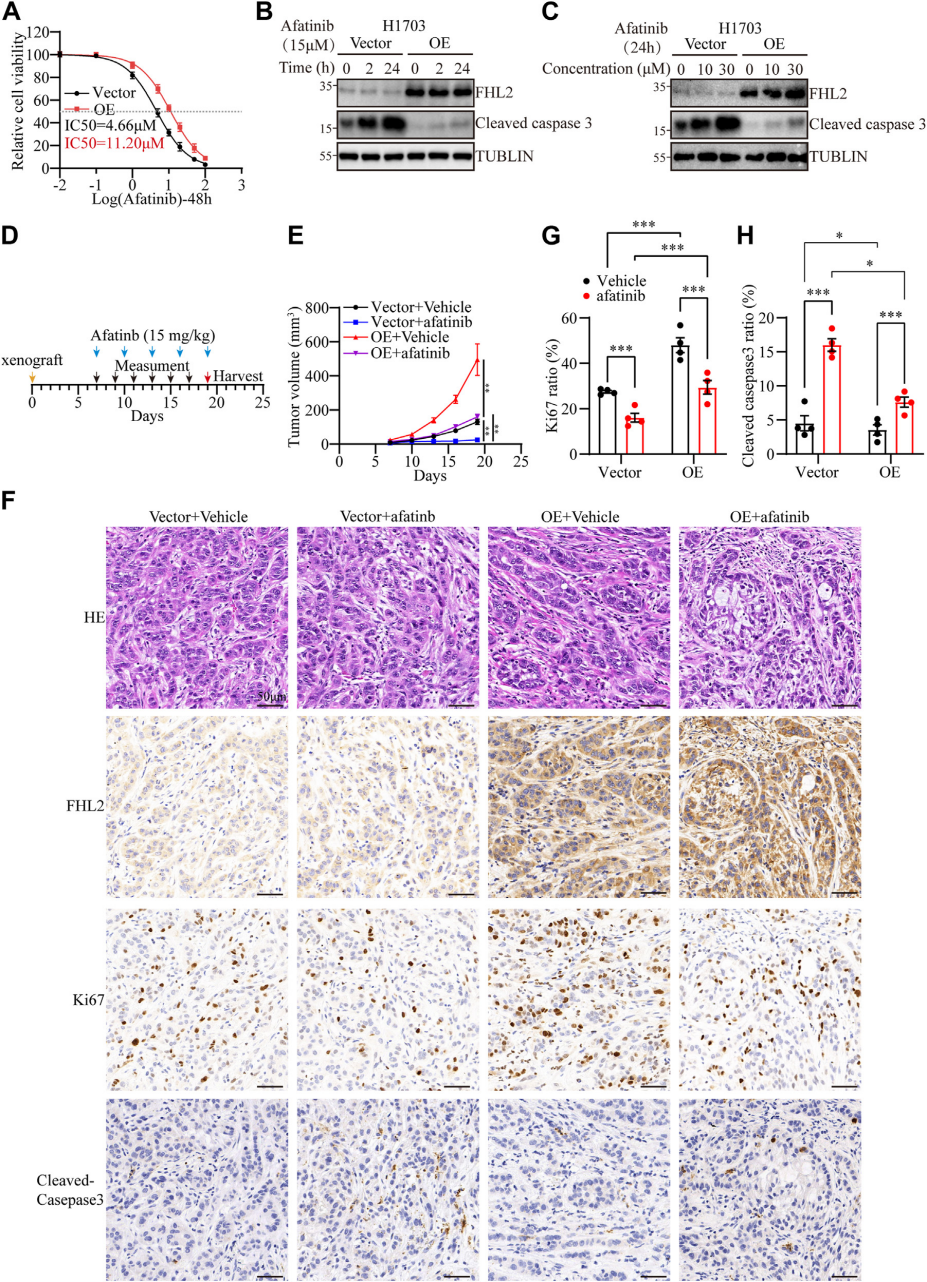
**FHL2 overexpression conferred resistance to afatinib**. *A*, IC50 of afatinib in H1703 cells at 48 h treatment. *B*, protein level of Cleaved-Caspase3 in H1703 cells after exposure to afatinib (15 μM) for different durations (0 h, 2 h, and 24 h). *C*, protein level of Cleaved-Caspase3 in H1703 cells treated with varying concentrations of afatinib (0 μM, 10 μM, and 30 μM) for 24 h. *D*, a scheme illustrating the treatment regimen for subcutaneous tumors inoculated with H1703 cells and administered afatinib. *E*, growth curve of subcutaneous tumors treatment with afatinib. *F*, representative images of IHC staining from subcutaneous tumor sections. *G*, quantification of KI67+ cell counts in the FHL2 overexpression and control groups after afatinib treatment. *H*, quantification of Cleaved-Caspase3+ cell counts in the FHL2 overexpression and control groups after afatinib treatment. The data were assessed with two-way ANOVA. ∗*p* < 0.05, ∗∗*p* < 0.01, ∗∗∗*p* < 0.001, ns: no significant. FHL2, four and a half LIM domains protein 2; IHC, immunohistochemistry.

Aberrant activation of the PAM signaling pathway is a critical factor contributing to resistance against TKIs (32). Our findings demonstrate that FHL2 overexpression upregulates PDK1 expression, leading to dysregulated activation of the PAM pathway. Based on this, we hypothesized that FHL2 overexpression might affect tumor cell sensitivity to afatinib. Supporting this, our IC_50_ assay revealed that FHL2 overexpression significantly attenuated the inhibitory effects of afatinib on H1703 cells, as indicated by an increased IC_50_ value (Fig. 6*A*). The time- and dose-dependent induction of apoptosis by afatinib was assessed in H1703 cells (Fig. 6, *B* and *C*). Notably, FHL2 overexpression significantly attenuated afatinib-induced apoptosis, further supporting its role in promoting resistance to afatinib therapy. Furthermore, the effect of afatinib treatment was evaluated in tumor xenograft models established with H1703 cells with FHL2 overexpression. A schematic representation outlines the treatment regimen for subcutaneous tumors (Fig. 6*D*). FHL2 overexpression significantly enhanced tumor growth rates in both vehicle-treated and afatinib-treated nude mice xenografts (Figs. 6*E* & Fig. S2*C*), suggesting that FHL2 promotes resistance to afatinib and facilitates tumor progression. IHC was conducted to examine the expression levels of FHL2, KI67, and Cleaved Caspase-3 in paraffin-embedded sections of the subcutaneous tumors (Fig. 6*F*). The results showed that FHL2 overexpression increased Ki67-positive cell numbers, indicating enhanced proliferation. In addition, afatinib treatment elevated Cleaved Caspase-3 levels, but this increase was mitigated in FHL2-overexpressing tumors, suggesting that FHL2 partially counteracts afatinib-induced apoptosis (Fig. 6*G*). These findings further suggest that FHL2 promotes tumor growth and attenuates the therapeutic efficacy of afatinib.

### Clinical cases show that LUSC patients with high FHL2 expression exhibit poor efficacy in response to afatinib treatment

**Figure 7:**
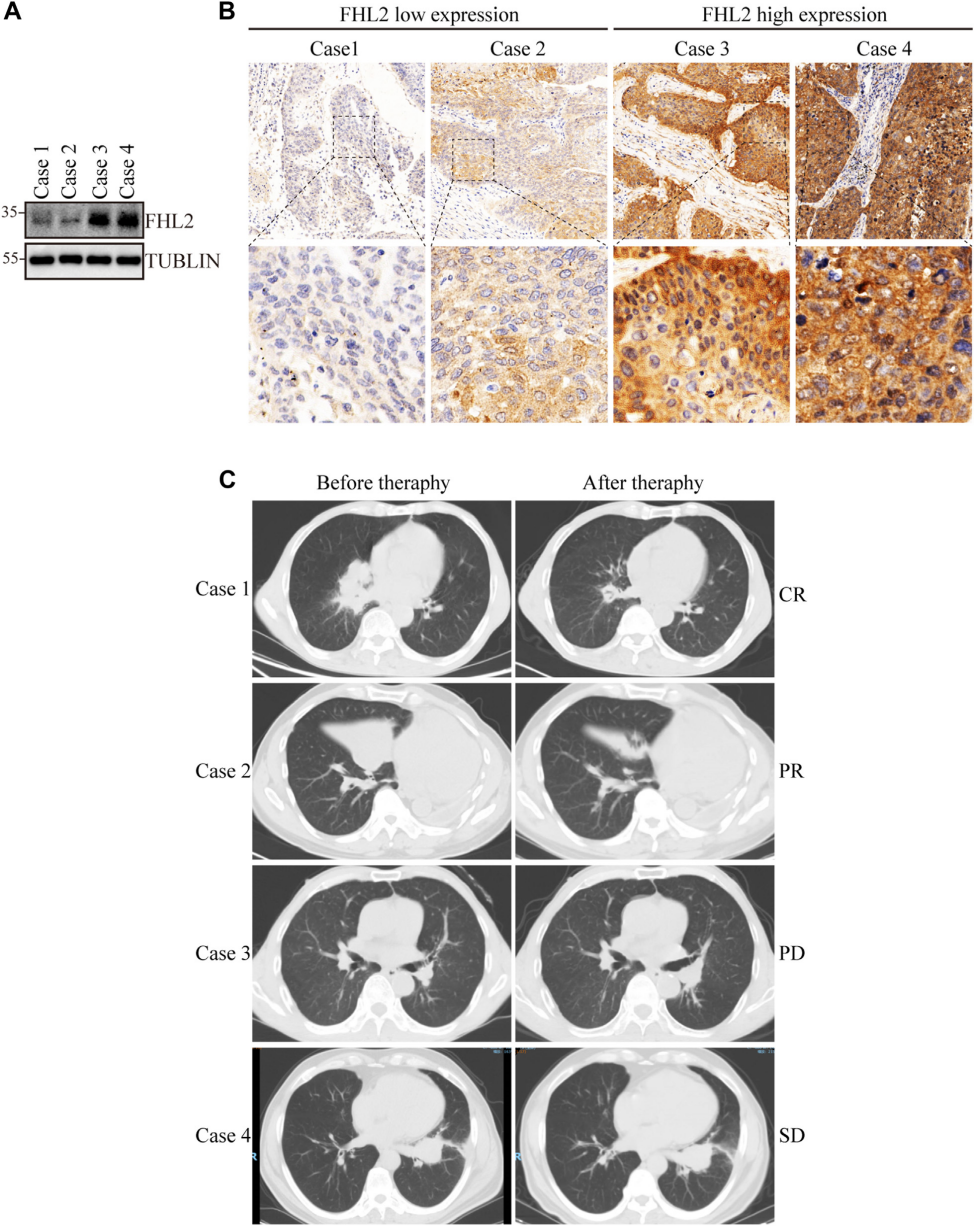
**LUSC patients with high FHL2 expression exhibit poor response to afatinib therapy**. *A*, WB analysis was performed to assess FHL2 levels in samples from four LUSC patients who underwent afatinib treatment. *B*, IHC staining was performed to validate FHL2 expression in the four LUSC samples. *C*, representative CT images of LUSC patients taken before and after afatinib therapy. CR, complete response; PR, partial respons; SD, stable disease; PD, progressive disease; LUSC, lung squamous cell carcinoma; FHL2, four and a half LIM domains protein 2; IHC, immunohistochemistry; WB, Western blotting; CT, computed tomography.

The association between FHL2 expression and the efficacy of afatinib therapy in four LUSC patients was evaluated using puncture specimens for protein extraction and paraffin block preparation. WB analysis (Fig. 7*A*) and IHC staining (Fig. 7*B*) revealed low FHL2 expression in cases 1 and 2, while cases 3 and 4 exhibited high FHL2 expression. Therapy efficacy, assessed by the response evaluation criteria in solid tumors (RECIST), showed partial response for cases 1 and 2, progressive disease for case 3, and stable disease for case 4 (Fig. 7*C*). These findings suggest that high FHL2 expression in LUSC patients is associated with poor efficacy of afatinib therapy.

## Discussion

**Figure 8:**
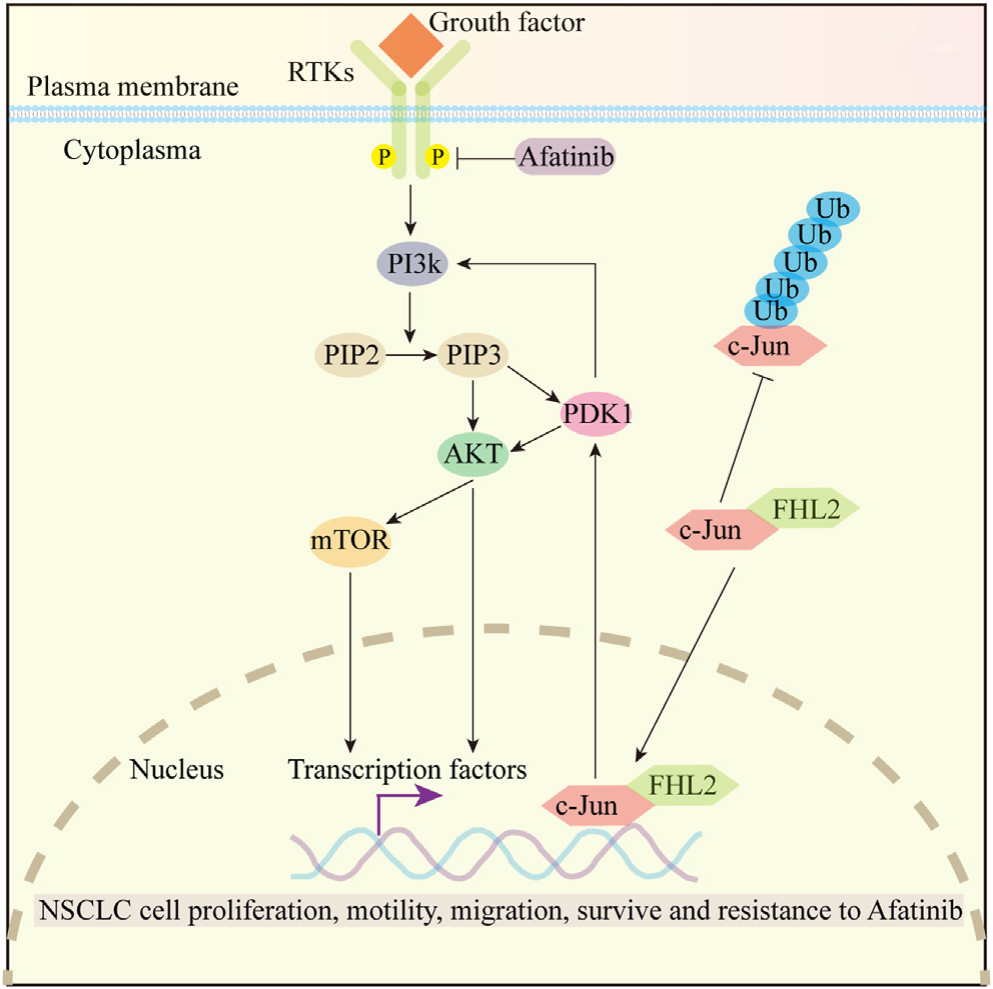
**A schematic representation illustrating how FHL2 orchestrates the growth and progression of LUSC by modulating key molecular pathways**. According to this model, FHL2 binds to c-Jun and suppresses its ubiquitination, thereby stabilizing the protein. As a transcriptional coactivator, FHL2 enhances c-Jun–mediated transcription of PDK1, leading to activation of the PAM signaling pathway, which in turn drives LUSC progression and contributes to afatinib resistance. LUSC, lung squamous cell carcinoma; FHL2, four and a half LIM domains protein 2; PDK1, 3-Phosphoinositide-dependent protein kinase 1; PAM, PI3K/AKT/mTOR.

In summary, our study underscores the critical role of FHL2 in driving LUSC progression and resistance to afatinib therapy. We propose a mechanistic model (Fig. 8) in which FHL2 directly binds to c-Jun, inhibits its ubiquitination and subsequent proteasomal degradation, thereby enhancing c-Jun protein stability. Functioning as a transcriptional coactivator, FHL2 promotes c-Jun–driven transcriptional upregulation of PDK1, ultimately resulting in aberrant activation of the PAM signaling pathway. This dysregulated activation contributes significantly to both the advancement of LUSC and the development of resistance to afatinib. Clinically, LUSC patients with high FHL2 expression exhibited the shortest OS and DFS. Moreover, FHL2 expression was identified as an independent prognostic factor for both OS and DFS. These findings provide valuable insights for researchers seeking novel prognostic biomarkers for LUSC and highlight FHL2 as a potential therapeutic target for afatinib-resistant LUSC patients.

FHL2 was initially identified in skeletal muscle cells and rhabdomyosarcoma (33, 34). Recent studies have indicated that FHL2 is widely expressed throughout the body, with particularly high expression in cardiac tissue (35). Pan-cancer analyses of FHL2 expression reveal a notable upregulation in certain malignant tumors, including CHOL, COAD, ESCA, HNSC, LUAD, LUSC, and PAAD. However, FHL2 expression is significantly reduced in other malignancies, such as GBM, KICH, LIHC, PCPG, PRAD, THCA, and UCEC) (Fig. 1*A*). In line with its expression patterns in tumors, high FHL2 expression is associated with poor OS in patients with THCA, CESC, KIRC, HNSC, LUAD, OV, and LUSC. Conversely, high FHL2 expression correlates with better OS in patients with LIHC, SARC, UCEC, THYM, and PCPG. (Fig. 1*D*). The dual roles of FHL2 as an oncogene or tumor suppressor in different cancer types suggest that its functional context is highly dependent on tissue type and interacting partners.

FHL2 is significantly upregulated in LUSC at both the mRNA level (Fig. 1, *A*–*C*) and the protein level (Fig. 2, *A* and *B*). Patients with high FHL2 expression exhibited the poorest OS and DFS (Fig. 1, *D* and *E* and Fig. 2, *F* and *G*). In addition, patients with larger tumors (>3 cm) or lymph node metastasis displayed significantly higher FHL2 levels compared to those with smaller tumors or no metastasis (Fig. 2, *C* and *D*). Multivariate analysis further identified FHL2 expression as an independent prognostic factor for both OS (HR: 1.470, CI: 1.093–1.978, *p* = 0.011) and DFS (HR: 1.545, 95% CI: 1.078–2.213, *p* = 0.018) (Table 2). These findings strongly suggest that FHL2 functions as an oncogene in LUSC and serves as a potential prognostic biomarker for this malignancy.

Recent studies have shown that FHL2 deficiency induces cell differentiation and suppresses gastric and colon tumorigenesis (9). FHL2 has been reported to function as a scaffold, enhancing TRIM63-mediated ubiquitination of APC, thereby activating the Wnt/β-catenin signaling pathway to promote LUAD progression (36). Moreover, previous research has shown that FHL2 promotes tumorigenesis in NSCLC by inducing angiogenesis and increasing vascular permeability (37). These findings highlight the critical oncogenic role of FHL2 in tumor development and progression. Notably, FHL2 exhibits diverse functions depending on the cellular context, acting as a multifunctional regulator involved in processes such as proliferation, migration, apoptosis, and signal transduction. In our study, gain- and loss-of-function experiments demonstrated that FHL2 promotes the proliferation, migration, invasion, and tumor growth of LUSC both *in vitro* and *in vivo* (Fig. 3, *H*–*W*). Mechanistically, bulk RNA-seq identified downstream genes and signaling pathways regulated by FHL2 (Fig. 4, *F*–*M*). Our findings revealed that FHL2 overexpression activates the PAM signaling pathway, whereas FHL2 knockdown suppresses this pathway by regulating PDK1 expression (Fig. 5, *A* and *B*). Furthermore, FHL2 interacts with c-Jun and inhibits its ubiquitination and proteasomal degradation, thereby stabilizing c-Jun protein (Fig. 5, *E*–*K*). Acting as a noncanonical coactivator of c-Jun, FHL2 promotes the transcriptional upregulation of PDK1, ultimately leading to the activation of the PAM signaling pathway. Previous research has demonstrated that FHL2 promotes the proliferation, migration, and invasion of LUAD cells by inhibiting autophagy through activation of the PAM signaling pathway (38). However, in cancer, autophagy plays a dual role—either suppressing or promoting tumor development—depending on factors such as tumor stage, genetic background, and the surrounding microenvironment (39). The role of FHL2 in regulating autophagy, however, remains incompletely understood and warrants further investigation.

Rescue experiments further confirmed that FHL2 regulates the PAM signaling pathway through the c-Jun/PDK1 axis (Fig. 5, *M*–*P*), and PDK1 is a critical downstream effector that sustains the oncogenic function in LUSC (Fig. S1, *A*–*C*). Furthermore, FHL2 overexpression inhibits apoptosis, while FHL2 knockdown promotes apoptotic activity (Fig. S4*D*), consistent with findings reported by Li *et al.* (40).

Afatinib, an irreversible ErbB-family inhibitor, is recommended as a first-line therapy for eligible patients with metastatic NSCLC harboring uncommon EGFR mutations, such as S768I, L861Q, and/or G719X (25, 41). The Phase III LUX-Lung 8 trial demonstrated promising outcomes for afatinib therapy in patients with metastatic LUSC (23). However, the clinical application of afatinib in LUSC is limited due to the low prevalence of EGFR-activating mutations (<5%) (42) and the emergence of acquired resistance. Aberrant activation of the PAM pathway has been identified as a key mechanism underlying afatinib resistance (43). Our study revealed that FHL2 overexpression upregulates PDK1 transcription *via* c-Jun activation. As a critical regulator of the PAM signaling pathway, PDK1 plays a pivotal role in this process. To further investigate the relationship between FHL2 expression and afatinib resistance, we conducted *in vivo* and *in vitro* experiments (Fig. 6, *A*–*G*). These experiments demonstrated that FHL2 overexpression significantly attenuates the inhibitory effects of afatinib on H1703 cells. Moreover, analysis of four clinical LUSC cases treated with afatinib confirmed that patients with high FHL2 expression exhibited poor therapeutic efficacy (Fig. 7, *A*–*C*). These findings strongly support the hypothesis that abnormally high FHL2 expression contributes to resistance to afatinib therapy, highlighting FHL2 as a potential biomarker for predicting afatinib efficacy in LUSC patients or as a potential therapeutic target.

The identification of FHL2 as a prognostic marker and potential therapeutic target in LUSC has significant clinical implications. Targeting FHL2 or its downstream effectors, such as PDK1 or the PAM pathway, may represent a novel strategy to overcome therapy resistance and improve patient outcomes. Future studies should explore the potential of combining FHL2 inhibition with current standard-of-care treatments, including immune checkpoint inhibitors and targeted therapies, to enhance therapeutic efficacy.

Although our study highlights the critical role of FHL2 in promoting LUSC progression and afatinib resistance *via* the c-Jun–PDK1–mTOR signaling axis, several limitations remain. First, the number of clinical LUSC samples treated with afatinib was relatively small, potentially limiting the statistical power and generalizability of the clinical findings. Second, while we confirmed the transcriptional regulation of PDK1 by the FHL2–c-Jun complex, further mechanistic investigations—such as mutational or structural studies—are needed to fully define this interaction. Third, a major limitation is the lack of genetic background information for the clinical specimens. Without data on key genomic alterations, such as EGFR, KRAS, or TP53 mutations, it is difficult to fully assess the relationship between FHL2 expression, afatinib resistance, and underlying oncogenic drivers. In future work, we plan to incorporate comprehensive genomic profiling to explore how FHL2 expression correlates with LUSC-relevant mutations and resistance mechanisms. These efforts may enhance the clinical relevance of FHL2 as a potential therapeutic target in EGFR-TKI–resistant LUSC.

## Conclusions

To sum up, our study reveals that FHL2 is a key regulator of LUSC progression and afatinib resistance through its modulation of the PAM pathway. These findings highlight the potential of FHL2 as both a prognostic biomarker and a therapeutic target in LUSC, offering new avenues for improving patient outcomes in this challenging malignancy.

## Experimental procedures

### Datasets

FHL2 expression data for NSCLC samples and normal lung tissues were retrieved from TCGA and the Genotype-Tissue Expression database, respectively. OS analysis for FHL2 in NSCLC patients was performed using the Kaplan–Meier Plotter platform (https://kmplot.com/analysis/). Pan-cancer analysis of FHL2 and coexpression gene analysis were performed using the TIMER 2.0 platform (http://timer.cistrome.org/). GSEA was performed using the GSEA software (V4.3.3). The heatmap was generated using an online platform for data analysis and visualization (https://www.bioinformatics.com.cn, last accessed on November 10, 2023) (44).

### Patients and follow-up

A total of 76 LUSC tissues and corresponding paracancerous tissues were collected from patients undergoing surgery at The Second Affiliated Hospital of Nanchang University between September 3, 2017, and September 26, 2018. Pathologic diagnoses were independently conducted by two pathologists following the World Health Organization's classification criteria. The final follow-up was conducted in December 31 2023. This study was approved by the Ethics Committee of the Second Affiliated Hospital of Nanchang University, and informed consent was obtained from all participants.

### TMA establishment and IHC assay

Tissue microarray (TMA) comprising 76 LUSC tissues and their corresponding paracancerous tissues were constructed by Shanghai Biochip Co, Ltd. IHC assays were conducted according to protocols detailed in our previous studies (45, 46) and detailed procedures are described in the Supplementary Methods and Materials. The expression of FHL2 was detected using a polyclonal antibody (1:3000, 21619-1-AP; Proteintech). The other primary antibodies were anti-cleaved caspase 3 (1:400, 9664S, CST), and anti-Ki67 (1:10000, 27309-1-AP, Proteintech). IHC results were quantified using the AHSQ online tool (https://lce.biohpc.swmed.edu/ahsq/analysis.php) (47). Select "Tumor" as the target cell region and "All" as the target cell structure. Diaminobenzidine intensity classification is as follows: negative (0–0.2), weak (0.2–0.4), moderate (0.4–1.0), and strong (>1.0).

### Cell line source

HBE cells, along with the BEAS-2B, A549, SK-MES-1, NCI-H1299, NCI-H1703, NCI-H1975, HCC827, and NCI-H226 cell lines, were used in this study. All cell lines were obtained from the Cell Bank of the Chinese Academy of Sciences.

### Cell culture conditions

Cells were cultured in Dulbecco's modified Eagle's medium or RPMI-1640 (HyClone) supplemented with 10% fetal bovine serum (HyClone) and 1% penicillin-streptomycin (Yeasen) at 37 °C in a humidified incubator with 5% CO_2_.

### Cell authentication and contamination testing

All cell lines were authenticated using short tandem repeat profiling and tested for *mycoplasma* contamination using the MycAway Plus-Color One-Step *Mycoplasma* Detection Kit (Yeasen) before use.

### Protein extraction and WB

Protein extraction and WB were performed following protocols described in our previous studies (45, 46). Tissue and cellular proteins for WB and IP assays were extracted using radioimmunoprecipitation assay (RIPA) buffer (PC101, Epizyme) supplemented with a protease inhibitor cocktail (GRF101, Epizyme). Protein concentrations were determined using a bicinchoninic acid protein quantification kit (ZJ101L, Epizyme).

Proteins were separated by SDS-PAGE and transferred onto polyvinylidene fluoride membranes (Millipore). Membranes were blocked for 1 h at room temperature (RT) using a protein-free rapid blocking buffer (PS108P, Epizyme) and incubated with primary antibodies overnight at 4 °C, followed by incubation with secondary antibodies for 1 h at RT.

The following primary antibodies were used: anti-FHL2 (1:1000, 21619-1-AP, Proteintech), anti-β-Tubulin (1:1000, 2128, CST), anti-P85 (1:1000, MA1-74183, Invitrogen), anti-p-P85 (1:1000, PA5-104853, Invitrogen), anti-P110α (1:1000, 4249S, CST), anti-P110β (1:1000, 3011S, CST), anti-PDK1 (1:1000, 18262-1-AP, Proteintech), anti-c-Jun (1:1000, 9165S, CST), anti-AKT (1:1000, 9272S, CST), anti-p-AKT (1:1000, 13038S, CST), anti-P70S6K (1:1000, 9202S, CST), anti-p-P70S6K (1:1000, 9234S, CST), anti-4EBP1 (1:1000, 9452S, CST), anti-p-4EBP1 (1:1000, 2855S, CST), anti-mTOR (1:1000, 2972S, CST), anti-p-mTOR (1:1000, 2971S, CST), anti-cleaved caspase 3 (1:1000,) anti-FLAG (1:1000, 20543-1-AP, Proteintech), anti-Bax (1:1000, 5023S, CST), anti-Ubiquitin (1:1000, 20326S, CST).

### Total RNA extraction and real-time polymerase chain reaction

Total RNA from cell lines and tissues was extracted with TRIzol reagent (Invitrogen) and reverse-transcribed into complementary DNA using the Hifair III 1st Strand cDNA Synthesis Kit (gDNA Digester Plus) (Yeasen), following the manufacturers' protocols. Quantitative real-time polymerase chain reaction was performed using Hieff qPCR SYBR Green Master Mix (Yeasen). GAPDH served as the internal reference gene. Relative RNA expression levels were calculated using the 2−ΔΔCt method. The primer sequences used in this study are provided in Table S2.

### Bulk RNA sequencing (RNA-seq)

Bulk RNA-Seq was conducted by OBiO Technology. Principal component analysis plots, volcano plots, and bubble plots were generated using RStudio (R version 4.3.0). GSEA was performed using the GSEA software (V4.3.3). Venn diagrams were created using the jvenn tool (https://jvenn.toulouse.inra.fr/app/index.html), and heatmaps were produced with the online platform https://www.bioinformatics.com.cn.

### Wound healing, Matrigel Transwell assay, plate colony formation assay and CCK8

The functional assays were conducted following protocols detailed in our previous studies. Comprehensive procedures are provided in the Supplementary Methods and Materials.

### Immunoprecipitation

Cells were lysed using RIPA lysis buffer (PC101, Epizyme) supplemented with a protease inhibitor cocktail (GRF101, Epizyme). A total of 800 μg of protein lysate was diluted with RIPA buffer to a final volume of 500 μl. The lysate was then incubated overnight with 50 μl of protein A/G magnetic beads (Epizyme) and 5 μl of anti-FHL2 antibody (21619-1-AP, Proteintech) or 2.5 μl of anti-IgG (30000-0-AP, Proteintech). To cross-link the immunoglobulins covalently to the protein A/G magnetic beads, 1 ml of freshly prepared 20 mM dimethyl pimelimidate in 0.2 M triethanolamine (pH 8.2) was added and incubated for 30 min at RT. The target protein was eluted with 2X SDS-PAGE loading buffer for 15 min at 95 °C. WB was performed to detect the results.

### Proximity ligation assay

Duolink In Situ PLA Probe Anti-mouse (DUO92004, Sigma-Aldrich) and Anti-Rabbit (DUO92002, Sigma-Aldrich) were utilized to perform PLA according to the manufacturer's instructions. In brief, cells were cultured on glass slides, followed by fixation with 4% paraformaldehyde (Yeasen) and permeabilization with 1% Triton-X-100. Afterward, the slides were blocked with a blocking solution for 60 min at 37 °C. Primary antibodies were applied and incubated overnight at 4 °C in a humidity chamber. The ligation step was performed using ligase for 30 min at 37 °C, followed by amplification with polymerase for 100 min at 37 °C. Fluorescent images were captured using a fluorescence microscope (Leica). The primary antibodies used in this experiment were anti-FHL2 (1:50, 21619-1-AP, Proteintech) and anti-c-Jun (1:50, MA5-15881, Invitrogen).

### CHX chase assay

To evaluate the effect of FHL2 on c-Jun protein stability, a CHX chase assay was performed. H1703 cells with FHL2 overexpression and SK-MES-1 cells with FHL2 knockdown were treated with CHX (50 μM, Sigma-Aldrich) to block new protein synthesis. Cells were harvested at 0-, 2-, 4-, 6-, 8-, and 10-h post treatment, followed by Western blot analysis to examine c-Jun protein levels over time.

### Transfection experiment

FHL2 lentiviral vectors were constructed by OBiO Technology (Shanghai). Transfection efficiency was evaluated using WB and qPCR. The shFHL2 sequences are provided in Table S3.

siRNA oligonucleotides were designed and synthesized by Tsingke. Transient transfections were performed using the Lipofectamine RNAiMAX Transfection Reagent (Invitrogen), following the manufacturer's instructions. The siRNA sequences are listed in Table S3.

### *In vivo* tumor growth

All animal experiments were approved by the Animal Experimentation Ethics Committee of The Second Affiliated Hospital of Nanchang University. Male BALB/c-nu/nu mice (4–6 weeks old) were purchased from CRISBIO (Shanghai) and housed in a specific pathogen-free environment.

For the xenograft models, approximately 5 × 10^6^ cells were resuspended in 150 μl of PBS and injected subcutaneously into the right flank of nude mice (6 mice per group). Tumor size was measured every 3 days, and tumor volumes were calculated using the formula (length × width^2^)/2. On the 19th day, the mice were euthanized, and tumors were harvested, fixed overnight in 4% paraformaldehyde, embedded in paraffin, and sectioned for HE and IHC staining.

In a separate experiment, BALB/c-nu/nu mice were injected subcutaneously with H1703-Vector or H1703-OE cells (5 × 10^6^ cells/mouse) and treated with afatinib (15 mg/kg, oral gavage, once every other day) starting on day seven for 2 weeks. Tumors were harvested on the 12th day after the first dose.

### Statistical analysis

Categorical variables were compared using Chi-squared or Fisher's exact tests, while statistical differences between groups were assessed with two-tailed Student's *t* tests or paired *t* tests. Two-way ANOVA was performed to analyze the effects of FHL2 overexpression and afatinib treatment on tumor size, Ki67 positivity, and Cleaved Caspase-3 positivity. Data are expressed as the mean ± standard error of the mean (SEM). The optimal cutoff value for the FHL2 H-score was determined using R. Kaplan–Meier survival analysis was conducted, and differences between survival curves were assessed using the log-rank test. Univariate and multivariate analyses were performed using two-sided Cox proportional hazards regression. A *p* value of <0.05 was considered statistically significant.

## Data availability

The data underlying this article will be shared on reasonable request to the corresponding author.

## Supporting information

This article contains supporting information.

## Ethics approval and consent to participate

The ethical approval for this study was provided by the Ethics Committee of the Second Affiliated Hospital of Nanchang University. Animal studies were conducted under the approved permit SYXX 2021–0004.

## Conflict of interest

The authors declare that they have no conflicts of interest with the contents of this article.

## Supporting information

Supporting information figure 1-4 legends

Raw data revision
