# Supplementary material for: FHL2 facilitates LUSC growth and therapy resistance through PI3K/AKT/mTOR activation
Source: J Biol Chem. 2025 Jun 6;301(7):110332. doi: 10.1016/j.jbc.2025.110332 (PMC12269609; doi:10.1016/j.jbc.2025.110332)
Supplement: Supplementary Methods and Materials [file mmc1.docx]

Immunohistochemistry (IHC)

Paraffin sections were heated at 60°C for 30 minutes and deparaffinized in three washes of xylene (5 minutes each). Sections were then rehydrated through a graded ethanol series: two washes in 100% ethanol for 10 minutes each, followed by sequential washes in 90%, 85%, and 75% ethanol for 10 minutes each. Antigen retrieval was performed by heating the sections in 1X citrate unmasking solution at 95–98°C for 10 minutes, followed by cooling on ice for 30 minutes.

Sections were blocked with 5% BSA (YESEN, China) at room temperature for 1 hour and then incubated with the primary antibody overnight at 4 °C. After washing three times with PBST (5 minutes each), endogenous peroxidase activity was blocked by incubating the slides in 0.3% H₂O₂ at room temperature for 30 minutes. Secondary antibody incubation was performed for 1 hour at room temperature.

The sections were stained with diaminobenzidine-H₂O₂ (Gene Tech) under a microscope, followed by counterstaining with hematoxylin. Finally, slides were sealed with neutral balsam (Yeasen, China) and cover slips. Negative control slides, processed without primary antibodies, were included in all assays.

Wound healing

Cells were seeded in six-well plates (Corning, USA) and cultured until they reached over 90% confluence. A straight line was scratched across the middle of each well using a pipette tip. The wells were washed three times with PBS to remove detached cells. Images were captured at 0 hours and 48 hours. The wound closure area was quantified using Image J software.

Matrigel Transwell Assay

The Matrigel invasion assay was conducted using 6.5 mm Transwell inserts (Corning, USA) with an 8.0 µm pore-size polycarbonate membrane pre-coated with Matrigel (0.5 µg/µL). A suspension of 1×10⁵ cells in serum-free medium was seeded into the upper chamber, while 500 µL of medium containing 10% FBS was added to the lower chamber as a chemoattractant. After 36 hours of incubation, the membranes were fixed in 4% paraformaldehyde for 10 minutes and stained with 0.4% crystal violet for 15 minutes. Non-invading cells on the upper surface of the membrane were gently removed. Images of stained cells on the lower surface were captured using a microscope, and cell counts were quantified with Image J software.

Plate Colony Formation Assay

The colony formation assay was conducted using 6-well plates. A total of 1000 cells were seeded per well in 3 mL of complete medium containing 10% FBS and cultured for 14 days to allow colony formation. The colonies were then fixed with 4% paraformaldehyde for 10 minutes and stained with 0.4% crystal violet for 15 minutes. Images of the stained colonies were captured using a digital camera.

CCK-8 assay

Cell proliferation was assessed using the CCK-8 assay. A total of 100 µL cell suspension was seeded into 96-well plates (5000 cells/well for H1703 and 2500 cells/well for SK-MES-1) and incubated at 37 °C with 5% CO₂ for 24, 48, 72, and 96 hours. Subsequently, 10 µL of CCK-8 solution (YESEN, China) was added to each well, followed by incubation for 90 minutes. Absorbance at 450 nm was measured after 24 hours to determine cell viability.

To evaluate afatinib-induced cytotoxicity, 100 µL of cell suspension was plated in 96-well plates at a density of 5000 cells/well for H1703 and 2500 cells/well for SK-MES-1, followed by incubation at 37 °C with 5% CO₂ for 24 hours. Afterward, 15 μM afatinib or an equivalent volume of DMSO was added to the wells and further incubated for an additional 24 hours. Cell viability was assessed by adding 10 µL of CCK-8 solution to each well, followed by a 90-minute incubation period. Absorbance was recorded at 450 nm to quantify cell viability. The formula for calculating cell viability is expressed as:

Viability (%) = [(A₄₅₀(Afatinib) - A₄₅₀(Blank)) / (A₄₅₀(DMSO) - A₄₅₀(Blank))] × 100.
