# Supplementary material for: FHL2 facilitates LUSC growth and therapy resistance through PI3K/AKT/mTOR activation
Source: J Biol Chem. 2025 Jun 6;301(7):110332. doi: 10.1016/j.jbc.2025.110332 (PMC12269609; doi:10.1016/j.jbc.2025.110332)
Supplement: Supplementary Tables 1-3 [file mmc2.docx]

Supplementary table 1 Antibody for western blotting, PLA, IP, and immunohistochemistry

| **Antibody** | **Company** | **Cat No.** |
| --- | --- | --- |
| FHL2 (Rabbit/IgG) | Proteintech | 21619-1-AP |
| c-JUN (Rabbit/IgG) | CST | 9165S |
| c-JUN (Mouse/IgG1) | Invitrogen | MA5-15881 |
| PDK1 (Rabbit/IgG) | Proteintech | 18262-1-AP |
| Cleaved Caspase-3 (Rabbit/IgG) | CST | 9664S |
| Ki-67 (Rabbit/IgG) | Proteintech | 27309-1-AP |
| p85 alpha (Mouse/IgG1) | Invitrogen | MA1-74183 |
| Phospho-PI3K p85 alpha (Tyr607)  (Rabbit /IgG) | Invitrogen | PA5-104853 |
| Akt (Rabbit) | CST | 9272S |
| Phospho-Akt (Thr308)  (Rabbit /IgG) | CST | 13038S |
| p70 S6 Kinase (Rabbit) | CST | 9202S |
| Phospho-p70 S6 Kinase (Thr389)  (Rabbit /IgG) | CST | 9234S |
| 4E-BP1 (Rabbit) | CST | 9452S |
| Phospho-4E-BP1 (Thr37/46)  (Rabbit /IgG) | CST | 2855S |
| mTOR (Rabbit) | CST | 2972S |
| Phospho-mTOR (Ser2448)  (Rabbit) | CST | 2971S |
| DYKDDDDK tag  (Rabbit /IgG) | Proteintech | 20543-1-AP |
| Peroxidase-Conjugated Goat Anti-Rabbit IgG(H+L) | YEASEN | 33101ES60 |
| Peroxidase AffiniPure Goat Anti-Mouse IgG (H+L) | YEASEN | 33201ES60 |
| IgG (Rabbit/IgG) | Proteintech | 30000-0-AP |
| β-Tubulin (Rabbit/IgG) | CST | 2128S |
| Bax | CST | 5023S |
| Ubiquitin (Rabbit/IgG) | CST | 20326S |
| P110α (Rabbit/IgG) | CST | 4249S |
| P110β (Rabbit/IgG) | CST | 3011S |

Supplementary table 2 The qRT-PCR primers used in this study

| **Gene** | **Forward primer (5’-3’)** | **Reverse primer (5’-3’)** |
| --- | --- | --- |
| FHL2 | ACTGGTGGACAAGCCCTTTG | CGTGGTGATGGGCTTTTTGC |
| JUN | CAGCCAGGTCGGCAGTATAG | GGACTCTGCCACTTGTCTCC |
| GAPDH | AGGTCGGAGTCAACGGATTTG | TGACAAGCTTCCCGTTCTCA |

Supplementary table 3 The sequence of shFHL2 and siRNA target

| **shRNA** | **Target sequence** |
| --- | --- |
| shFHL2-1 | CGACTGCTTTAACTGTAAGAA |
| shFHL2-2 | CCAATTGGAACCAAGAGTTTC |
| shFHL2-3 | CCCAAAGACAATCAGAATTTC |
| sh-NC | CCTAAGGTTAAGTCGCCCTCG |
| siPDK1 | 5' -GUACUUUUUGGAUCGAUUCUACATG- 3' |
|  | 5' -CAUGUAGAAUCGAUCCAAAAAGUACUG- 3' |
| si-JUN-1 | 5'- AGCAUUCUUGUCACAAUAAAUGUAT- 3' |
|  | 5'-AUACAUUUAUUGUGACAAGAAUGCUGU-3' |
| si-JUN-2 | 5'- UCACUCUCAGUGCUUCUUACUAUTA- 3' |
|  | 5' -UAAUAGUAAGAAGCACUGAGAGUGAUU- 3' |
