# Supplementary material for: FHL2 facilitates LUSC growth and therapy resistance through PI3K/AKT/mTOR activation
Source: J Biol Chem. 2025 Jun 6;301(7):110332. doi: 10.1016/j.jbc.2025.110332 (PMC12269609; doi:10.1016/j.jbc.2025.110332)
Supplement: Supporting information figure 1-4 legends [file mmc3.docx]

**Supplementary figure legends**

Fig.S1 FHL2 expression in LUAD

A. FHL2 expression in LUAD and normal lung tissues from the TCGA and GTEx databases. Data are presented as mean ± SD.

B. FHL2 expression in 49 paired LUAD and normal lung tissues from TCGA, analyzed using a paired t-test.

C. Analysis of FHL2 expression and OS in LUAD from TCGA data.

D. Heatmap displaying the expression of core genes in the PI3K/AKT signaling pathway from the TCGA database.

E&F. Correlation analysis of FHL2 and DDR2, SNAIL1 expression from the TIMER 2.0 database.

*****P* < 0.0001

Fig.S2 Representative images of subcutaneous xenograft tumors.

A&B. Representative images of subcutaneous xenograft tumors generated using SK-MES-1 and H1703 cells with FHL2 knockdown or overexpression.

C. Representative images of subcutaneous xenograft tumors derived from H1703 cells with FHL2 overexpression, following treatment with afatinib.

Fig.S3 Bubble plots showing the results of GO analysis.

A&B. Bubble plots displaying GO analysis results in H1703 and SK-MES-1 cells with FHL2 overexpression or knockdown.

Fig.S4 Functional experiments were conducted to investigate the effect of PDK1 knockdown in H1703-OE cells.

A. Proliferation ability was assessed using the CCK-8 assay in H1703-OE cells with PDK1 knockdown.

B. Wound healing assay was performed to evaluate the migration of H1703-OE cells with FHL2 knockdown, with quantification shown.

C. Matrigel transwell assay was conducted to evaluate the invasion of H1703-OE cells with FHL2 knockdown, with quantification shown.

D. To investigate the effect of FHL2 on apoptosis-related proteins, Bax and cleaved caspase-3 expression was detected in H1703 and SK-MES-1 cells via WB.
